# Core transcription factors, Oct4, Sox2 and Nanog, individually form complexes with nucleophosmin (Npm1) to control embryonic stem (ES) cell fate determination

**DOI:** 10.18632/aging.100222

**Published:** 2010-11-12

**Authors:** Helena Johansson, Stina Simonsson

**Affiliations:** Department of Medical Biochemistry and Cell Biology; Institute of Biomedicine; University of Gothenburg, box 440, 405 30 Gothenburg, Västra Götaland; Sweden

**Keywords:** Npm1, Oct4, Sox2, Nanog, ES cells, Tpt1

## Abstract

Embryonic stem (ES) cells have therapeutic potential in regenerative medicine, although the molecular mechanism controlling their pluripotency is not completely understood. Depending on interaction partners most proteins can be involved in several different cellular mechanisms. We screened for novel protein-protein interactions using *in situ* proximity ligation assays together with specific antibodies directed against known important ES cell proteins. We found that all three core transcription factors, namely Oct4, Sox2 and Nanog, individually formed complexes with nucleophosmin (Npm1). We showed that the Npm1/Sox2 complex was sustained when cells were induced to differentiate by retinoic acid, while decreased in the other differentiation pathways. Moreover, Oct4 also formed individual complexes with translationally controlled tumor protein (Tpt1). Downregulation of *Npm1* or *Tpt1* increased mRNA levels for genes involved in mesoderm and ectoderm differentiation pathways, respectively, indicative of their involvement in ES cell maintenance. We have here described four novel protein-protein interactions in ES cell involving all three core transcription factors. Our findings improve the current knowledge about ES cell-specific protein networks and indicate the importance of Npm1 and Tpt1 to maintain the ES cell phenotype.

## INTRODUCTION

Embryonic stem (ES) cells possess the capacity of unlimited self-renewal while maintaining pluripotency. Their ability to differentiate into all cell types of the three embryonic germ layers makes them interesting candidates for cell replacement therapies. Much effort has been put into understanding what makes these cells unique. This has led to the identification of three core transcription factors that are essential for maintenance of ES cells: Oct4, Sox2 and Nanog [1]. Considerable effort has also been invested in attempts to dedifferentiate somatic cells towards pluripotency, a strategy that could be used for personalized regenerative medicine. One approach is to virally induce exogenous expression of transcription factors, forming induced pluripotent stem (iPS) cells. These iPS cells, which are similar to ES cells in morphology, proliferation, and capacity to form teratomas, were first generated from mouse fibroblasts by retroviral induction of four transcription factors: Oct4, Sox2, c-Myc, and Klf4 [2]. The same factors were found to have the same type of dedifferentiating effects in several human cell lines and in addition, different sets of transcription factors with this effect have been identified [3-6]. Dedifferentiation procedures have so far shown very low efficiency. This depend partly on the cell differentiation stages of the original cells, where aging cells have a barrier for reprogramming and acquisition of immortality is a crucial and rate-limiting step for successful development of iPS cells [7]. Protein content in the original somatic cells may also affect the efficiency but still no further proteins have been assigned important for the iPS cell creation.

Nucleophosmin/nucleoplasmin family member 1 (Npm1, also known as B23, Numatrin or NO38) is expressed at high levels in mouse [8] and human [9] ES cells. It is a multifunctional phosphoprotein that has been implicated in cell proliferation [10] as well as regulation of transcription, where it appears to be able to both repress [11] or stimulate [12-13] transcription. We recently showed that Npm1 and translationally controlled tumor protein (Tpt1, also referred to as TCTP, Fortilin, Histamine-releasing factor HRF, or P23) form a complex in ES cells and that this complex is important for cell proliferation [14]. Tpt1 has previously been shown to improve reprogramming efficiency of somatic cell nuclear transfers [15], which is another method for dedifferentiation of somatic cells. As previously stated, cell proliferation has been shown to be a criteria for successful iPS cell creation and Npm1 has also previously been shown to interact with one of the four factors for iPS cell creation i.e. c-Myc [16]. In view of these findings, we here investigated if Npm1 and Tpt1 network with other factors identified as important in iPS cell creation and ES cell maintenance.

## RESULTS

### Oct4 interacts physically with Npm1 and Tpt1 in ES cells

**Figure 1.**
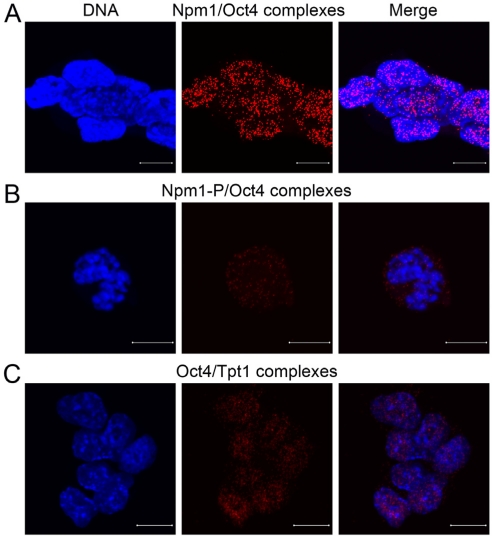
Oct4 physically interacts with Npm1 and Tpt1 in ES cells. Immunofluorescence confocal microscopy in combination with in situ PLA, which detects protein-protein complexes, was used to explore interactions between Oct4 to Npm1 and Tpt1. Each detected complex is represented by a red dot. DNA was counterstained by Hoechst 33342 (blue). Scale bar represents 10 μm. (**A**) Complexes between endogenous Npm1 and Oct4 were found in the nucleoplasm of interphase cells. (**B**) Complexes between Npm1 and Oct4 during mitosis using an antibody specific to phosphorylated Npm1. (**C**) Complexes between endogenous Oct4 and Tpt1 in the nucleoplasm of interphase cells.

Oct4 is needed for maintenance of ES cells and iPS cell creation, but its relation to Npm1 and Tpt1, two factors found to be important for ES cell proliferation, have not been addressed and remained elusive. *In situ* proximity ligation assay (PLA) [17] is a powerful tool to screen rather easily for protein-protein interactions. Confocal micrographs collected at 0.38 μm intervals and merged together, show high number of Npm1/Oct4 complexes in the nucleoplasm of interphase ES cells (Figure 1A, each red dot represents one detected interaction). Interaction was also observed in mitotic cells using an antibody only recognizing Npm1 phosphorylated at residue T198 (Figure 1B, red dots). Oct4 also formed individual complexes with Tpt1 and a considerable number of Oct4/Tpt1 complexes are seen in the nucleus of interphase ES cells (Figure 1C, red dots).

In brief, both Npm1 and Tpt1 physically interact individually with Oct4 in ES cells, and the interactions are not cell cycle dependent.

### Npm1 physically interacts with Sox2 in ES cells

**Figure 2.**
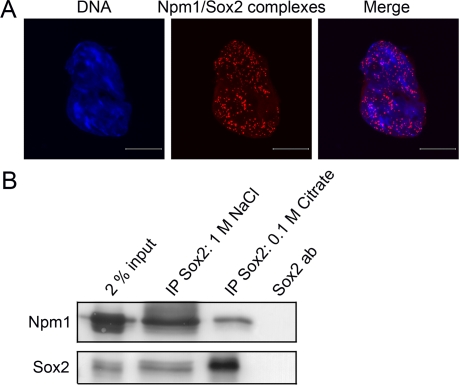
Sox2 physically interacts with Npm1 in ES cells. (**A**) Immunofluorescence confocal microscopy in combination with in situ PLA showed that there is an interaction between Sox2 and Npm1 in ES cells. Complexes (red dots) were detected in the nucleoplasm of interphase cells. DNA was counterstained by Hoechst 33342 (blue). Scale bar represents 10 μm. (**B**) Co-immunoprecipitation experiments followed by Western blot analysis showed that Npm1 can be immunoprecipitated using anti-Sox2 (1 M NaCl and 0.1 M Citrate).

In addition to Oct4, Sox2 is another of the three important core transcription factors identified in ES cells. Using *in situ* PLA the possible interaction of Sox2 with Npm1 and Tpt1 was investigated. Confocal micrographs collected at 0.38 μm intervals and merged together, showed a substantial number of Npm1/Sox2 complexes in the nucleus of interphase cells (Figure 2A, red dots). The samepattern was observed with another set of Npm1/Sox2 antibodies (anti-Sox2 [MAB2018, R&D Systems] and anti-Npm1 [ab15440, abcam]; data not shown).

To further verify these results, extract prepared from ES cells was subjected to co-immunoprecipitation with anti-Sox2 followed by Western blot. Npm1 was co-immunoprecipitated with anti-Sox2 (Figure 2B, IP Sox2: 1 M NaCl and 0.1 M Citrate) but not with IgG control (data not shown).

No interaction was observed between Tpt1 and Sox2 and was therefore used as one of the negative controls for the *in situ* PLA method (Suppl. Figure 1). The PLA together with co-immunoprecipitation establishes that endogenous Npm1 physically interacts with endogenous Sox2 in ES cells.

### Npm1/Sox2 interaction changes during differentiation

**Figure 3.**
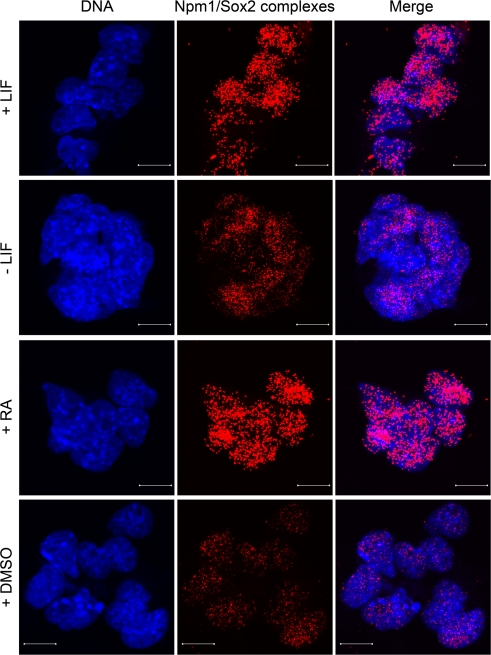
Npm1/Sox2 interaction changes during differentiation. Induced differentiation of ES cells by withdrawal of leukemia inhibitory factor (LIF), addition of either dimethyl sulfoxide (DMSO) or retinoic acid (RA) for 24 h in combination with *in situ* PLA, showed that the Npm1/Sox2 complexes decreased during DMSO and leukemia inhibitory factor withdrawal induced differentiation. In contrast, retinoic acid induced differentiation did not notably affect the number of Npm1/Sox2 complexes in the nucleoplasm. DNA was counterstained with Hoechst 33342 (blue). Scale bar represents 10 μm.

Both Sox2 and Npm1 protein levels are changing when ES cells start to differentiate. To investigate how the interaction is affected in the beginning of differentiation, ES cells were treated toward different differentiation pathways (retinoic acid, dimethyl sulfoxide and withdrawal of leukemia inhibitory factor) in combination with *in situ* PLA. Npm1/Sox2 complexes were shown to decrease when differentiation was induced by dimethyl sulfoxide or withdrawal of leukemia inhibitory factor (Figure 3). Conversely, such complexes remained, or even increased in number when differentiation was induced by addition of retinoic acid. This analysis showed that the Npm1/Sox2 interaction is reduced during conditions known to induce differentiation of ES cells into mesoderm and endoderm, whereas differentiation by retinoic acid into ectodermal lineage is induced in the continuous presence of Npm1/Sox2 complexes.

### Npm1 also interacts with the third core transcription factor, Nanog

As stated in the introduction, three core transcription factors, namely Oct4, Sox2 and Nanog, have been proven essential for ES cell maintenance. We have now shown that Npm1 form protein-protein complexes with both Oct4 and Sox2, which prompted us to investigate how the interactions are between Nanog, Npm1 and Tpt1, using *is situ* PLA in combination with confocal microscopy.

**Figure 4.**
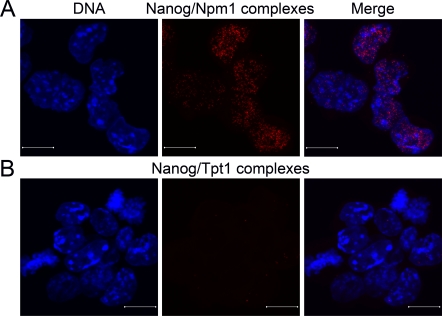
Npm1 and Nanog interact in ES cells. Immunofluorescence confocal microscopy in combination with *in situ* PLA, which detects protein-protein complexes, was used to explore interactions between Nanog to Npm1 and Tpt1. Each detected complex is represented by a red dot. (**A**) Complexes between endogenous Npm1 and Nanog were found in the nucleoplasm in some of the interphase ES cells. (**B**) No interaction was observed between Tpt1 and Nanog in ES cells. DNA was counterstained by Hoechst 33342 (blue). Scale bar represents 10 μm.

Confocal micrographs collected at 0.38μm intervals and merged together, showed that Nanog interacts with Npm1 (Figure 4A, red dots), whereas no interaction was seen between Nanog and Tpt1 (Figure 4B).

These analyses conclude that endogenous Npm1 interacts with all three core transcription factors in ES cells since it also interacts with endogenous Nanog.

### Tpt1 and Npm1 are involved in the differentiation of ES cells

**Figure 5.**
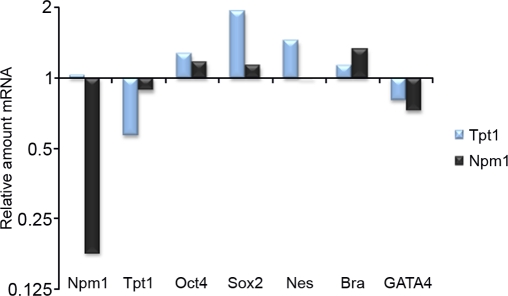
Tpt1 and Npm1 are involved in ES cell maintenance. Involvement of Tpt1 and Npm1 in ES cell maintenance was investigated using shRNA constructs against each gene and analyzed by qPCR 48 h post transfection. Downregulation of *Tpt1* (blue bars) resulted in a minor increase in *Oct4* and *Brachyury* (Bra) levels, a decrease in *GATA4* levels, whereas levels of *Sox2* and *Nestin* (Nes) increased significantly, indicative of involvement in ectodermal differentiation. Downregulation of *Npm1* (black bars) resulted in minor increases in *Oct4* and *Sox2* levels, decreased *GATA4* levels, while increased *Brachyury* (Bra) levels, indicative of involvement in mesodermal differentiation. All experiments were done in triplicates and normalized first to *GAPDH* (reference gene), and then to corresponding negative control shRNA constructs.

ES cells have the capacity to differentiate into all three germ layers: endoderm, mesoderm and ectoderm. We studied whether shRNA mediated downregulation of *Tpt1* or *Npm1* affected the ES cell maintenance analyzed by qPCR. As shown in Figure 5, shRNA mediated downregulation of *Tpt1* gave a minor increase in the of *Oct4* levels, whereas levels of *Sox2* and the ectodermal differentiation marker *Nestin* was increased (blue bars). *Npm1* downregulation neither affected levels of *Oct4*, nor *Sox2* notably, but increased the mesodermal marker *Brachyury* (black bars).

qPCR data from shRNA mediated downregulation of *Npm1* and *Tpt1* respectively, indicates that both proteins seem to be involved in different differentiation pathways.

## DISCUSSION

Before iPS stem cells can be used in regenerative medicine several obstacles have to be overcome. One of these is the poor efficiency of the process. While some cell types readily are dedifferentiated, other cells are resistant to the same viral treatment. Tpt1 have previously been implicated in regulation of Oct4 in somatic cell nuclear transfers [18], but somewhat contradictory, our shRNA knockdown of *Tpt1* in ES cells did not notably affect *Oct4* transcription, if any effect it was a slight increase in *Oct4* levels. A possible explanation of this opposite effect could be that we only had a 50% decrease of *Tpt1* and that the remaining amount of Tpt1 was enough to keep *Oct4* normal. We show that Tpt1 interacts with Oct4, while no interaction was found between Tpt1 and Sox2, or together with Nanog. The interaction between Tpt1 and Oct4 may be associated with the finding that Tpt1 increases Oct4 levels in somatic nuclear transfers [18], since Oct4 is known to self-regulate [19]. Interestingly, *Sox2* and the ectodermal differentiation marker *Nestin* increased during downregulation of *Tpt1*. In support of our findings, Tpt1 protein levels decrease during neural cell differentiation [20]. Other reports also implicate a role for Tpt1 in embryonic development. Knockout mice deficient in both *Tpt1* alleles are embryonic lethal and depending on if the entire gene [21] or part of the gene [22] is deleted, they die around E3.5 and E9.5, respectively. E3.5 is around the blastocyst stage from which ES cells are propagated from the inner cell mass of the developing embryo [23]. Altogether, these results imply that Tpt1 is required for ES cell maintenance as well as involved in ectoderm lineage formation.

Further, we found that Npm1 interacts with all three core transcription factors, namely Oct4, Sox2 and Nanog in ES cells. The Npm1/Oct4 interaction is ES cell specific due to the fact that Oct4 is an ES cell specific protein which becomes downregulated when the cells differentiate [24]. Available data regarding Npm1 and ES cells show that Npm1 is needed for ES cell proliferation, but neither affect levels of Oct4 or Nanog, nor differentiation [25]. This is in accordance with our shRNA downregulation of *Npm1* which did not notably affect levels of neither *Oct4* nor *Sox2*. Sox2 is crucial for ES cells. However, it is still expressed after differentiation and it has been shown to be an important protein for development of epiblast and extraembryonic ectoderm [26]. Therefore the Npm1/Sox2 interaction was investigated during different paths of differentiation. By withdrawal of leukemia inhibitory factor or addition of dimethyl sulfoxide the number of complexes and their intensity were decreased, while the number of complexes was stable or even slightly increased by addition of retinoic acid. This implies that Npm1/Sox2 has a function not only in ES cells but also functions in ectodermal cells, at least during the first stages of differentiation. This is supported by our finding that *Npm1* depletion increased the expression of mesodermal marker *Brachyury* and did not affect the ectodermal marker *Nestin*. Suppression of Npm1 in neural stem cells inhibits cell proliferation, induces apoptosis through the p53 pathway but does not affect cell differentiation [27]. This may imply that the Npm1/Sox2 interaction has a role in pushing ES cells towards ectodermal differentiation, but that once they have started to differentiate into neural stem cells, they can differentiate further without Npm1 involvement. Knockout mice deficient in both *Npm1* alleles are embryonic lethal. The mice show aberrant organogenesis and die around E11.5 owing to severe anemia resulting from defects in primitive haematopoiesis [28]. Thus, Npm1 seems to have several different important functions during the embryonic development, and further studies are needed to explore these roles.

Nanog is to some extent very different from the other two core transcription factors. Heterogeneous expression of Nanog is observed in ES cells [29] and overexpression of Nanog is enough to keep ES cell maintenance in the absense of LIF [30]. Nanog has also recently been implicated in G1 to S transition, where Nanog overexpression results in quicker cell cycle progression through accelerated S-phase entry by direct binding and regulation of two proteins important for this process [31]. We have previously shown that overexpression of Npm1 also results in higher cell proliferation rates [14], so our newly found interaction between Nanog and Npm1 might very well play a part in cell cycle regulation. Although, given that Npm1 shows individual interactions with all three core transcription factors, it argues also for a role in transcriptional regulation. Previously it has been shown that Npm1 functions as a histone chaperone that remodels local chromatin structures [32]. Therefore one logical explanation for these three interactions would be that Npm1 remodels the chromatin structure so that the different transcription factors can bind and activate the specific genes.

We have shown that both Npm1 and Tpt1 are involved in ES cell maintenance and they both form individual complexes with Oct4. Npm1 also forms complexes with Sox2 and Nanog and the Npm1/Sox2 interaction are sustained in the early parts of ectodermal differentiation. Since Npm1 interacts with several factors identified necessary for iPS cell creation, it may has a critical role for successful dedifferentiation. Especially since cell proliferation has been shown to be a crucial criterion for successful iPS cell creation and Npm1 is essential for cell proliferation in both ES [14,25], neural stem cells [27] and in other cell systems [10].

## MATERIALS AND METHODS

### Cell cultures

Cell lines were grown at 37°C in humidified atmosphere containing 5% CO_2_. Murine ES cell line R1 was maintained on mitomycin C inactivated mouse embryonic fibroblasts in Dulbecco's modified eagle's medium supplemented with 15% fetal calf serum, 1.0 mM sodium pyruvate. 0.1 mM nonessential amino acids, 2.0 mM L-glutamine, 0.1 mM β-mercaptoethanol, 100 U/100 μg penicillin/streptomycin, 20 mM Hepes pH 7.3, and 1000 U/ml leukemia inhibitory factor (ESGRO, Chemicon).

### Cell extracts

Whole cell extracts were prepared by harvesting confluent cell cultures containing approximately 3×10^7^ cells. Harvested cells were incubated in ice cold extraction buffer (10 mM Tris-HCl pH 7.4, 10 mM MgCl_2_, 10 mM KCl, 1.0 mM DTT) containing protease inhibitor cocktail tablet (Complete, Roche Diagnostics) for 10 min at 4°C. The addition of NP40 to 1%(v/v) was followed by incubation for 10 min, 4°C. Cell lysates were homogenized and NaCl was added to a final concentration of 420 mM followed by incubation for 1 h, 4°C. The extracts were cleared by centrifugation (19,000×*g*, 1 h, 4°C) and the supernatant was frozen and stored in liquid nitrogen.

### Generic *in situ* proximity ligation assay

3-4×10^4^ cells were grown on chamber slides overnight. Differentiation was induced by subtraction of leukemia inhibitory factor, incubation with 2.0 μM retinoic acid or 2 % dimethyl sulfoxide for 24 h. Cells were fixed in 4% paraformaldehyde/PBS for 20 min, permeabilized with 0.25% Triton X-100/PBS for 5 min, and blocked in 10% fetal calf serum in 0.1% Triton X-100/PBS for 20 min. Primary antibody (anti-HRF/Tpt1 [M099-3, Clone 6E9, Nordic Biosite]; anti-Npm1 [ab15440 or ab10530, Abcam]; anti-Oct4 [611203, Clone 40, BD Biosciences]; anti-Sox2 [ab15830, Abcam]; anti-Nanog [RCAB0002P-F, Cosmo Bio Co., Ltd]) diluted in 0.1% Triton X-100/PBS/1% fetal calf serum were added for 2 h. Duolink (Olink Biosciences) *in situ* proximity ligation assay (PLA) was performed according to the manufacturer's protocol. PLA probes were diluted in 0.1% Triton X-100/PBS/1% fetal calf serum and incubated in a pre-heated humidity chamber for 1 h at 37°C, followed by hybridization, ligation, amplification and detection. The distance between the two primary antibodies needs to be less than 40 nm to generate a signal in this assay, making the methodology highly specific for physically interacting protein-protein complexes. Slides were analyzed using an inverted Zeiss LSM 510 META confocal microscope equipped with a Zeiss image processing system. An 63×/1.4 NA oil objective and sequential scanning with narrow band-pass filters was used (420-480 nm for Hoechst 33342 and 560-615 nm for Alexa 613).

### Co-immunoprecipitation

Co-immunoprecipitation was performed with Dynabeads Protein G (Invitrogen) according to the manufacturer's protocol by addition and crosslinking with dithiobispropionimidate-2HCl of 10 μg anti-Sox2 [ab15830, Abcam] or normal rabbit IgG antibody [sc-2027, Santa Cruz]. Approximately 0.2 mg of whole cell extract was incubated with the antibody-beads overnight, 4°C. Proteins were eluted in 1 M NaCl (50 mM Tris pH 7.5, 1.0 M NaCl, 0.1% NP40, 1.0 mM DTT) with the use of the magnet. Extended elution was done with 0.1 M Citrate with the use of the magnet. Elutes were mixed with 2x Laemmli buffer and heated to 95° for 5 min and analyzed by Western blot.

### Western blot

Proteins were separated using SDS-PAGE, followed by semi-dry electrotransfer onto polyvinylidene difluoride membranes for 1 h, 100 mA/gel in transfer buffer (48 mM Tris, 39 mM Glycin, 1.3 mM SDS, 10% methanol) and immunologically detected. Membranes were blocked with 5% skim milk in PBS containing 0.1% Tween 20 for 1 h and incubated with primary antibody (anti-Npm1 [ab10530, Abcam]; anti-Sox2 [ab15830, Abcam]) in blocking solution overnight at 4°C. After washing with PBS-Tween, blots were incubated with secondary antibody (AP conjugated goat anti-mouse IgM+IgG+IgA (H+L); AP conjugated goat anti-rabbit IgM+IgG (H+L chain specific) [Southern Biotechnology Associates]) in blocking solution for 1 h at room temperature. Visualization of proteins was done with BCIP/NBT kit (Invitrogen).

### Short hairpin RNAs and transfection

Short hairpin RNAs (shRNA) were obtained from SABiosciences. Preparation of shRNA was done according to the manufacturer's protocol. Npm1 clone 3: GGC AGA AGC AAT GAA CTA T, with puromycin selection and Tpt1 clone 1: GAG CTG CAG AGC AGA TTA, with GFP tag were used at concentration of 0.4-1 μg plasmid. Equal amounts of negative shRNA control: GGA ATC TCA TTC GAT GCA TAC with either puromycin selection or GFP tag was used. 4-5x10^4^ cells were transfected with Lipofectamine LTX (Invitrogen) 4 h post seeding according to the manufacturer's standard protocol. 48 h post transfection mRNA levels were analyzed using quantitative RT-PCR (qPCR).

### Quantitative RT-PCR

Total RNA was isolated from shRNA transfected cells using RNeasy mini kit with the addition of RNase-Free DNase to eliminate contaminating genomic DNA (Qiagen). 1 μg of RNA was subjected to reverse transcription into cDNA using SuperScript III (Invitrogen), according to the manufacturer's protocol. Endogenous gene copy numbers were determined by qPCR analysis using the ABI PRISM 7900 system with SYBR Green mix reagents (Applied Biosystems). Briefly, the qPCR mixture contained 1 μl cDNA, 1×SYBR Green mix reagent and 50 nM of each primer in a total reaction volume of 20 μl. All samples were analyzed in triplicates using the primer pairs listed in Table 1. Each primer pair yielded a single product, confirmed by dissociation curve analysis, and gave no product in the no-template control. To analyze the obtained data, all samples were normalized to an internal reference gene (GAPDH) to eliminate sample variances and toward a sample transfected with a vector containing a nonsense shRNA construct to eliminate effects from transfection.

**Table 1.** Primer sequences used for qPCR analysis

Genes	Primer sequences (Forward/Reverse)
**Tpt1**	5' TGATCATCTACCGGGACCTCA 3' 5' GCGATCTCCCGGATCTTGTA 3'
**Npm1**	5' CTTACGGTTGAAGTGTGGTTCA 3' 5' TCATCATCATCCTCATCATCCT 3'
**Oct4**	5' CACGAGTGGAAAGCAACTCA 3' 5' AGATGGTGGTCTGGCTGAAC 3'
**Sox2**	5' CACAACTCGGAGATCAGC 3' 5' CTCCGGGAAGCGTGTACTTA 3'
**Nestin**	5' AAAGGAAAGGCAGGAGTCCCTGAA 3' 5' TGGTCCTCTGCGTCTTCAAACCTT 3'
**Brachyury**	5' AGCTCTCCAACCTATGCGGACAAT 3' 5' TGGTACCATTGCTCACAGACCAGA 3'
**GATA4**	5' ACTCCAAAGTGCTGGGTTCAATGC 3' 5' TTGCAGAGGGTAGATGTTCAGGCT 3'
**GAPDH**	5' AATGTGTCCGTCGTGGATCTGA 3' 5' GATGCCTGCTTCACCACCTTCT 3'

Following primer pairs were used to measure endogenous mRNA levels of proteins of interest, regarding ES cell maintenance (Oct4, Sox2), ectodermal differentiation (Nestin, Sox2), mesodermal differentiation (Brachyury), endodermal differentiation (GATA4) and reference gene (GAPDH) for internal normalization, after shRNA mediated downregulation of Tpt1 or Npm1.

## SUPPLEMENTARY DATA

Supplementary Figure 1. *in situ* proximity ligation assay controls. (**A**) Positive control for in situ PLA. Oct4 and Sox2 are two highly important transcription factors in ES cells that are known to interact [Okumura-Nakanishi S, Saito M, Niwa H, Ishikawa F. Oct-3/4 and Sox2 regulate Oct-3/4 gene in embryonic stem cells. J Biol Chem 2005; 280:5307-17]. Using anti-Oct4 (Oct-3/4 (H-134): sc-9081, Santa Cruz) and anti-Sox2 (MAB2018, Clone 245610, R&D Systems), *in situ* PLA is able to detect Oct4-Sox2 complexes and confirm that the method is working correctly. (**B**) Negative control to visualize in situ PLA background staining using anti-Tpt1 (HRF (FL-172): sc-30124, Santa Cruz) and anti-Sox2 (MAB2018, Clone 245610, R&D Systems) which do not interact with each other (top row) or no primary antibodies (bottom row) were used. The absence of red dots in these experiments shows the high specificity of this method. DNA was counterstained by Hoechst 33342 (blue). Scale bar represents 10 μm.
