# Supplementary figures and images for: Core transcription factors, Oct4, Sox2 and Nanog, individually form complexes with nucleophosmin (Npm1) to control embryonic stem (ES) cell fate determination
Source: Aging (Albany NY). 2010 Nov 12;2(11):815–22. doi: 10.18632/aging.100222 (PMC3006024; doi:10.18632/aging.100222)

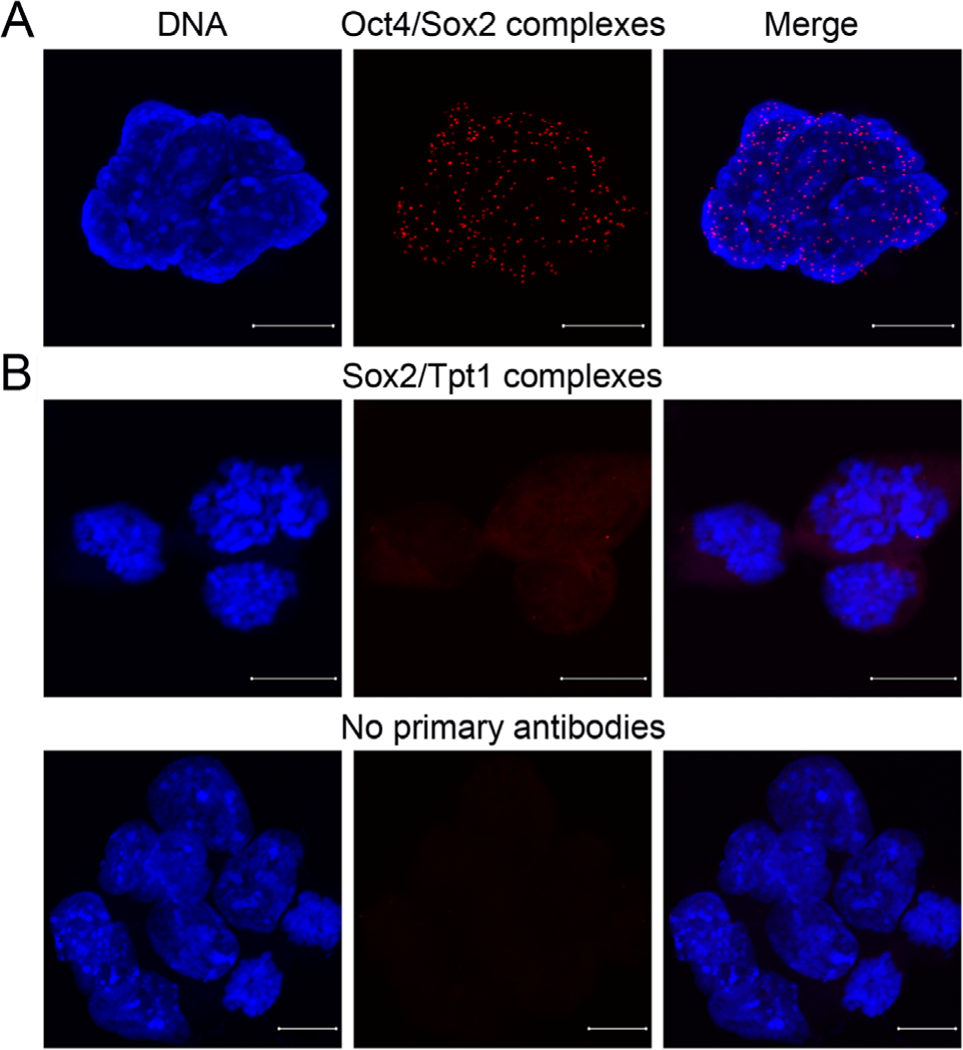

Supplement: Supplementary Figure 1. — (A) Positive control for in situ PLA. Oct4 and Sox2 are two highly important transcription factors in ES cells that are known to interact [Okumura-Nakanishi S, Saito M, Niwa H, Ishikawa F. Oct-3/4 and Sox2 regulate Oct-3/4 gene in embryonic stem cells. J Biol Chem 2005; 280:5307-17]. Using anti-Oct4 (Oct-3/4 (H-134): sc-9081, Santa Cruz) and anti-Sox2 (MAB2018, Clone 245610, R&D Systems), in situ PLA is able to detect Oct4-Sox2 complexes and confirm that the method is working correctly. (B) Negative control to visualize in situ PLA background staining using anti-Tpt1 (HRF (FL-172): sc-30124, Santa Cruz) and anti-Sox2 (MAB2018, Clone 245610, R&D Systems) which do not interact with each other (top row) or no primary antibodies (bottom row) were used. The absence of red dots in these experiments shows the high specificity of this method. DNA was counterstained by Hoechst 33342 (blue). Scale bar represents 10 μm. [file aging-02-815-s001.tif]
